# Development of an Ex Vivo Assay for Identification of Infectious Hepatitis E Virus in Different Kinds of Food Samples

**DOI:** 10.3390/pathogens12101231

**Published:** 2023-10-11

**Authors:** Renate W. Hakze-van der Honing, Sophie van Oort, René A. M. Dirks, Wim H. M. van der Poel

**Affiliations:** 1 Wageningen Bioveterinary Research (WBVR), Wageningen University and Research, Houtribweg 39, 8221 Lelystad, The Netherlands; sophie.vanoort@wur.nl (S.v.O.); wim.vanderpoel@wur.nl (W.H.M.v.d.P.); 2 Wageningen Food Safety Research (WFSR), Wageningen University and Research, Akkermaalsbos 2, 6708 Wageningen, The Netherlands

**Keywords:** Hepatitis E virus, HEV, cell culture, infectivity, primary hepatocytes

## Abstract

Hepatitis E virus (HEV) is a positive-sense single-stranded RNA virus and a major cause of acute viral hepatitis. HEV is responsible for 20 million infections worldwide in humans every year. HEV-3 and HEV-4 are zoonotic and are responsible for most of the HEV cases in developed countries. Consumption of contaminated pig meat or pig products is considered to be the main transmission route of HEV HEV-3 in Europe. Prevalence studies for HEV generally use PCR methods to detect the presence or absence of genomic RNA. However, these methods do not discriminate infectious virus particles from non-infectious material. Previously developed HEV cell culture systems only worked with high efficiency after cell line adaptation of the subjected virus strains. In this manuscript, the development of a culture system for the detection of infectious HEV strains is described. For this purpose, we optimized the isolation and the growth of primary hepatocytes from young piglets. Subsequently, the isolated hepatocytes were used to culture HEV of different origins, such as liver tissue samples and sausage samples. This method can be applied to better assess the risk of infection through consumption of food products associated with HEV RNA contamination.

## 1. Introduction

Hepatitis E virus (HEV) is a positive-sense single-stranded RNA virus and a major causative agent of acute viral hepatitis. HEV is responsible for 20 million infections worldwide in humans every year [1]. HEV is a member of the Hepeviridae family, which comprises eight different HEV genotypes. Five of these genotypes, HEV-1 to HEV-4 and HEV-7, can infect people [2]. HEV-1 and HEV-2 are associated with human-to-human transmission via faecal–oral contamination of water and food and are responsible for endemic outbreaks in developing countries. HEV-3, HEV-4, and HEV-7 are zoonotic and HEV-3 and HEV-4 are responsible for most of the HEV cases in developed countries. HEV-3 has been found in humans, domestic swine, wild boar, deer, rabbit, and mongoose and is widely distributed around the world, while HEV-4 is mainly detected in Asia in humans, domestic swine, and wild boar [3,4]. HEV-7 has been found in camels and was detected in an immunocompromised patient [5]. HEV infection can cause liver inflammation, fever, and jaundice in humans and chronic HEV infections have been reported in immunocompromised individuals [3,6,7]. The majority of infections are self-limiting, 3 out of 20 million infections are acute cases. Around 57,000 cases per year are fatal [1].

Consumption of contaminated pig meat or pig products is considered to be the main transmission route of HEV HEV-3 in Europe [8], although a comprehensive overview of transmission route(s) is lacking. The food-borne transmission route of HEVs has been supported by epidemiological and virological findings. For example, Colson et al., in a report about consuming raw pig liver sausage [9], demonstrated of the infectiousness of HEV in pork sausages and pork livers [10,11].

Pigs can also shed HEV into the environment, where the virus can stay infectious for a longer period [12]. Such an environmental spread may lead to the contamination of drinking water and the contamination of crops [13]. In The Netherlands, 17% of surface water samples were demonstrated to be HEV RNA positive in a 2009 study [14]. In European countries, more than 50% of the pig farms may be affected and HEV seroprevalence within these farms can be over 80% [15].

Prevalence studies for HEV and other viruses generally use PCR methods to detect the presence or absence of HEV specific genomic sequences. However, these methods do not discriminate infectious virus particles from non-infectious material. The development of cell culture assays that can provide in vitro proof of infectivity has proven challenging for many viral pathogens. Hepatitis E virus culture has been performed using several cell lines, such as A549, PLC/PRF/5, and HepG2/C3A [16,17,18,19,20], in which specific HEV viruses were propagated. Most HEV cell culture systems only worked with high efficiency after cell line adaptation of the subjected virus strains. To this date, no quick HEV culture system is available that can be used to provide proof of infectivity for a broad range of HEV strains from different kinds of food matrices, environmental samples, or blood products.

In this paper, we describe the development of a culture system for the detection of infectious HEV field stains in a range of food matrices. For this purpose, we optimized the isolation and growth of primary hepatocytes obtained from young piglets. Subsequently, these isolated hepatocytes were used to culture HEVs of different origins, such as liver tissue samples and sausage samples.

## 2. Materials and Methods

### 2.1. Isolation and Growing of Primary Hepatocytes

The pig livers (experiment number 2020.D-0031.021) were obtained under the legislation of the Dutch Central Authority for Scientific Procedures on Animals (CCD license no. AVD40100202010965) and as such approved by The Netherlands animal welfare body and the Wageningen University and Research animal welfare body, in compliance with EU legislation.

**Figure 1:**
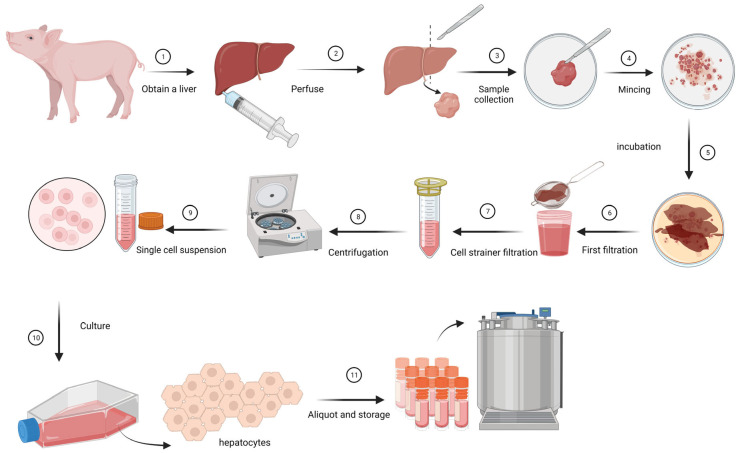
Schematic overview of isolation and growth of primary hepatocytes. (1) Purchase of a liver from a piglet. (2) Perfusion of the liver with DMEM/F12 (Gibco) medium to dispose of blood. (3) Sample collection. (4) Tissue mincing, cutting liver tissue into small pieces (around 2 square mm). (5) Incubation with 1% collagenase during 30 min at 37 °C. (6) Filtration of liver tissue using a tea strainer to get rid of larger lumps of liver. (7) Second filtration step using a cell strainer to end up with single cells. (8) Centrifugation and rinsing of the hepatocytes pellet. (9) Resuspending of the hepatocyte pellet in DMEM/F12. (10) Culture the hepatocytes until a 90–100% full monolayer can be observed. (11) Aliquoting and storage in liquid N2 until use. Created with BioRender.com. https://www.biorender.com/ (accessed on 5 July 2023).

To collect primary liver cells, fresh livers obtained from young piglets of 3–9 weeks old, were perfused with Dulbecco’s Modified Eagle Medium/Nutrient Mixture F-12 (DMEM/F12) (Gibco, Landsmeer, The Netherland,) with 1% Anti/Anti (Gibco) until the blood was flushed out of the tissue. Single-cell suspensions were created by mincing liver tissue into small pieces of 5–5 mm size, incubation in 0.1% collagenase IV in DMEM/F12 with 1% Anti/Anti (Gibco) for 1 h at 37 °C, followed by straining using a 70 μm cell strainer. The cell suspensions were centrifuged for 5 min at 250× *g* and pellets were washed with DMEM/F12 (Gibco) with 1% Anti/Anti (Gibco). The liver cells were cultured in a growth medium containing DMEM/F12 with 10% foetal bovine serum (FBS), 1% anti/anti (Gibco), and 40 µL/mL Primary Hepatocyte Maintenance Supplements (Gibco) in a T150 culture flask coated with Collagen I, rat tail (Corning, Amsterdam, The Netherlands,). Before culturing the cells in the T150 flasks, they were incubated with 12 mL 100 µg/mL Collagen 1 in 0.02 M acetic acid. After two hours the flasks were washed with PBS and dried for 4 h or used directly. The next day the cells were washed stringently to get rid of cells other than hepatocytes. As soon as the cells had been growing confluent for five to seven days, these cells were ready to be used in inoculation experiments or were stored in liquid nitrogen for later use (Figure 1). Cells were harvested by with 0.5% Trypsin (Gibco), and after washing and concentrating they were stored in DMEM/F12 medium with 10% Dimethyl sulfoxide (DMSO) (Thermo Fisher Scientific, Landsmeer, The Netherlands), and 10% FBS at a concentration of 1 million hepatocytes per vial.

### 2.2. Immune Peroxidase Monolayer Assay (IPMA)

The obtained hepatocytes were identified macroscopically. To confirm that the collected cells were predominantly hepatocytes, an identification test using a hepatocyte nucleus antibody (anti-HNF4A, Aviva Systems Biology, San Diego, CA, USA) was performed. Confluent monolayers of hepatocytes were washed with DPBS and dried before being frozen at −80 °C. Thereafter, the IPMA was performed. Hepatocytes were fixated for 30 min with 4% paraformaldehyde. The paraformaldehyde was removed and the cells were washed twice using a washing buffer (PBS+ 0.05% Tween20). The cells were permeabilized by 10 min incubation with 1% Triton followed by two washes using washing buffer (PBS+ 0.05% Tween20). The cells were incubated for one hour with anti-HNF4A 1:200 in blocking buffer 1 (4% horse sera in HISbuffer (WUR)) [18] followed by a second antibody goat anti-rabbit Horseradish peroxidase (HRP) conjugate (Dako, Leuven, Belgium) 1:500 in blocking buffer 2 (4% horse sera in conjugate buffer); in between and after incubations the cells were washed three times with washing buffer. This was followed by a 20 min incubation in AEC mix (1 mL 50 mM Sodium acetate, 50 µL AEC (3-Amino-9-ethylcarbazole) and 2.5 µL H_2_O_2_). As soon as the colour reaction was displayed, the AEC mix was replaced with washing buffer and cells were examined by phase-contrast microscopy.

### 2.3. Inoculation of Primary Hepatocytes

All the inoculation experiments were performed twice, in individual experiments at different time points. In each experiment a negative (non-infected hepatocytes) and a positive control (a HEV PCR-positive hepatocyte sample) was added.

One day before inoculation, 1 million hepatocytes were seeded onto 6 wells plates in growth medium, resulting in a 70% confluent monolayer the next day. Subsequently, the hepatocytes were inoculated with a 1 ml positive control sample (Table 1). After incubation for 1.5 h, the inoculate was removed and cells were washed 3 times with PBS (Gibco), prior to adding 2 mL growth medium. Every 2nd or 3rd day 50% of the medium was refreshed. For optimization experiments, starting at D0, after removing the inoculum at the start of the experiment, and every refresh step, a supernatant sample and cell fraction sample was collected. For this experiment, 5 wells were infected with exactly the same sample. For each sample moment a well was used. Both sample types, supernatant and cell fraction, were analysed by HEV real-time RT-PCR [21]. Prior to the RT-PCR an RNA isolation was performed using the Quick-DNA/RNA viral MagBead kit according to the manufacturer’s manual. After the final optimization of the cell culture system, supernatant samples were only collected and tested by HEV real-time RT-PCR after removing the inoculum at the start of the experiment (day 0) and on day 6 or 7. To prove the viability of the HEV obtained from the cell culture, supernatants collected on day 6 of the procedure were transferred to new hepatocytes and tested for HEV RNA positivity at D0 and D6 using real-time RT-PCR.

Several liver samples from retail, liver samples from previous animal experiments, a faecal sample, and a sausage sample from a study by Berto et al. [10] of a Ct value around or below 30 (approximately 10^4^ genome copies/mL) in the HEV RT-PCR were tested in the optimized infectivity assay (Table 1). Archived samples or samples from previous animal experiments had been tested again by HEV RT-PCR before they were used in the infection assay. Samples with a Ct under 30 were used in the infection assay. All such samples had been stored at −80 °C. A 10% homogenate of cells from disrupted organ tissue was prepared in culture medium without serum. The tissue was homogenized 1 min with the use of the Ultra-Turrax^®^ Tube Drive (IKA, Staufen, Germany). This homogenate was centrifugated at 4 °C for 15 min at 4600× *g* to pellet the tissue remnants and 1 mL of the supernatant was used to inoculate the hepatocytes. For processed samples, such as from sausages, that required extra purification, a 0.8/0.2 µM filtration step Thermo Fisher Scientific (Landsmeer, The Netherlands,) was performed according to the manufacturer’s manual. The inoculation and the cell culture propagation were performed as described above. Supernatant samples were taken at D0 and D6-7 and tested by HEV real-time RT-PCR [21].

### 2.4. RNA Extraction/Isolation and Real-Time RT-PCR

RNA isolation was carried out using the Quick viral DNA/RNA kit (Zymo Research, Irvine, CA, USA); in each isolation run a negative control (PBS) and positive control (HEV positive control Table 1) was added. A volume of 150 μL sample was added to 150 μL DNA/RNA shield (Zymo Research, Irvine, CA, USA) and extracted according to the manufacturer’s manual or stored at −80 until isolation. The DNA/RNA was eluted in 50 μL elution buffer. HEV RNA was amplified on the LC480 (Roche diagnostic, Almere, The Netherlands) machine with the real-time RT-PCR targeting the ORF2 of HEV described by [21] using the TaqMan Fast Virus 1-step Master Mix (Applied Biosystems, Bleiswijk, The Netherlands). The concentration of the positive samples was identified with a calibration line of a HEV positive sample with a known copy number. The concentration of this positive sample was determined with the WHO HEV standard.

### 2.5. Sensitivity of the System Compared to a Pig Model

The study (experiment number 2020.D-0037.002) was performed under legislation of the Dutch Central Authority for Scientific Procedures on Animals (CCD license no. AVD40100202114785) and approved by the Animal Welfare Body of Wageningen University and Research prior to the start of the in-life phase.

Two groups of each 6 SPF pigs at the age of 8 weeks were intravenously inoculated with 2 mL HEV-positive liver suspension and one group was infected with a HEV negative liver suspension. The first group was inoculated with a 10% liver suspension containing around 10^7^ copies/mL and the second group was inoculated with circa 10^4^ copies/mL, the concentration of the inoculum was determined using a calibration line of a HEV positive sample with a known copy number. The concentration of this positive sample was determined using the WHO HEV standard. The 10^7^ copies/mL suspension was diluted 1000× in a negative 10% liver suspension to obtain the 10^4^ copies/mL inoculation. We have chosen the 10^4^ copies/mL concentration because this corresponds to a Ct value of Ct30. The pigs were tested twice a week during a 4-week period, using a HEV-specific RT-PCR assay [21] for the presence of HEV in the faecal swabs. The faecal swabs were proceeded in 2 mL medium of which 150 µL was tested. Simultaneously, primary hepatocytes were inoculated in duplicate with the same samples at concentrations 10^7^, 10^4^, 5 × 10^3^, and 2.5 × 10^3^ copies/mL. A sample of the supernatant of the culture was taken at D0 and at D6 of the experiment and tested using a HEV-specific RT-PCR assay [21].

## 3. Results

### 3.1. Isolation and Culturing of the Primary Hepatocytes

**Figure 2:**
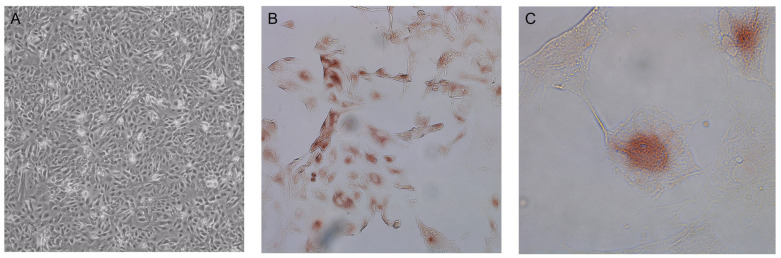
(**A**) Hepatocytes monolayer, cultured in collagen-coated flasks. (**B**) (10×) Hepatocytes grown in a monolayer. (**C**) (40×) Hepatocytes grown in a monolayer; the red colouring shows the nucleus labelled with anti-HNF4A.

The primary hepatocytes which were isolated from the fresh livers of young piglets were monitored daily for at least one week by microscopic inspection until a monolayer was formed (Figure 2A). In the microscope observation we identified mainly hepatocytes and a few fibroblasts. To confirm the presence of hepatocytes, which we attempted to isolate, we labelled them with hepatocyte-specific anti-HNF4A (Figure 2B,C). Only of the hepatocytes the nucleus were coloured red in the IPMA. The fibroblasts were not labelled with the anti-HNF4A.

**Figure 3:**
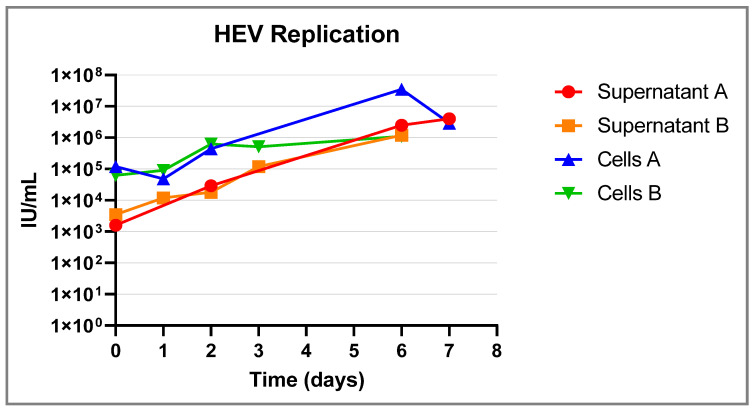
Hepatocytes were inoculated with an HEV-positive sample at D0. Subsequently, and at specific time points (marked with a dot), a supernatant sample and a cell-fraction sample were taken. The experiment was performed twice at different time moments. The samples of both experiments were tested in an HEV real-time RTPCR assay to detect HEV RNA. Results are plotted in the graph.

To prove the susceptibility of the primary hepatocytes, samples were inoculated with known HEV-positive samples. The supernatant and cell fraction of the cell culture were tested with a HEV-specific real-time PCR at several time points, and in both a reduction in the Ct value was demonstrated (Figure 3). When we transferred the supernatant to new hepatocytes, we again observed a propagation of HEV particles (HEV RT-PCR copy number increase).

A cytopathic effect was observed at none of the time points during the culturing of HEV in primary hepatocytes. After 6–7 days of propagation, the increase in HEV copy numbers stopped. Therefore, we ended all the incubations at day 6–7.

After optimization of the hepatocyte culture system, the system was inoculated with various naturally contaminated HEV RT-PCR positive samples such as liver samples from previous animal experiments, sampled in retail stores, and a sausage sample from a study by Berto et al. [10] (Table 1). In these naturally contaminated HEV samples we could measure an increase in HEV copies at the end of the propagation period; this increase demonstrates that our in vitro HEV infectivity assay successfully allows for replication of HEV strains from a variety of origins.

### 3.2. Sensitivity of the System Compared to the Animal Pig Model

Two groups of 6 piglets were infected with, respectively, 2 mL 10^7^ copies/mL and 10^4^ copies/mL. All of the animals excreted HEV in their faeces (Table 2). In the cell culture, we infected the hepatocytes, respectively, with 10^7^, 4 × 10^5^, 8 × 10^4^, 10^4^, 2 × 5 × 10^3^, and 2.5 × 10^3^ in duplicate and all the dilutions showed replication which could be deduced from the decrease in Ct value (Table 2).

**Table 2:** Comparison of the primary hepatocytes infection model with the animal model. Hepatocytes were infected with an HEV-positive liver of various concentrations. At D0 and D6 a sample was taken and tested in an HEV real-time RT-PCR to detect HEV RNA. Replicating virus was identified by demonstration of an increase in copy numbers at D6 compared to the amount of HEV precent at D0 after washing away the inoculum. Two groups of 6 piglets were infected with, respectively, 107 copies/mL and 104 copies/mL. Twice a week faecal samples were tested for the presence of HEV RNA by real-time PCR.

Concentration of the Inoculum (Genomic Copies/mL)	Concentration of the Inoculum (Log Genomic Copies/mL)	Log Genome Copies/mL at D0	Log Genome Copies/mL at D6	Primary Hepatocytes Model (Increase Log Copies/mL at D6 after Inoculation)	Animal Model (Total Animals/Tested Positive)
10^7^	7.00	1.29 (SD = 0.00)	5.14 (SD = 0.12)	3.47 (SD = 0.66)	6/6
4 × 10^5^	5.60	1.50 (SD = 0.30)	4.32 (SD = 0.01)	3.52 (SD = 0.38)	Nt
8 × 10^4^	4.90	1.30 (SD = 0.02)	4.32 (SD = 0.01)	3.02 (SD = 0.03)	Nt
10^4^	4.00	1.68 (SD = 0.46)	3.33 (SD = 0.06)	1.65 (SD = 0.52	6/6
3.33 × 10^3^	3.52	1.29 (SD = 0.00)	2.73 (SD = 0.73)	1.45 (SD = 0.34)	Nt
2 × 10^3^	3.30	1.29 (SD = 0.00)	2.16 (SD = 0.15)	0.87 (SD = 0.15)	Nt

Nt: not tested.

## 4. Discussion

To assess the risk of HEV infection after the consumption of HEV RNA-positive food items, a method to demonstrate the viability of HEV in food products was needed. Currently, animal experiments, in which HEV RNA-positive food products are inoculated into susceptible animals, also provide this information. However, animal experiments are ethically undesirable and expensive; therefore, they have limited use, necessitating the need for a broadly applicable in vitro HEV infectivity assay. Other research has suggested capsid integrity assays to assess virus infectivity, for example using RNase treatment followed by RNA extraction and RT-PCR [25]. However, RNA may also be protected against RNAse in case of a damaged capsid, potentially resulting in an overestimation of HEV infectivity in a sample when a virus with a damaged capsid is not infectious anymore. A cell culture-based infectivity assay combines a direct demonstration of the infectivity of HEV in a product (as in a pig model) with the possibility of greatly increased throughput, reduced cost of measurements, and reduced ethical concerns [12].

In previous research, HEV was cultured on several cell lines, such as A549, PLC/PRF/5, and HepG2/C3A [16,17,18,19,20,26]. These systems were often time-consuming, used only for specific HEV strains, or tested with just one of two strains. Moreover, such systems may only work after cell line adaptation of the selected HEV strains. For samples from the field, that have not adapted to the selected cell lines, such testing systems may not work and therefore may be less useful compared to the ex vivo system described in this study. Lately, Chew et al., optimized the previously described PLC/PRF/5 cell culture system [27] and came to higher titres than in previous studies. However, in their study they only tested one HEV-4 strain and one rat strain and a long culture time of the cells prior to the infection is still needed.

To develop a culture system to demonstrate HEV infectivity in field samples from different food matrices, we optimized the isolation and the growth of primary hepatocytes obtained from young piglets. For a successful hepatocyte isolation, a rich medium (DMEM/F12 with 10% foetal bovine serum (FBS), 1% anti/anti (Gibco), and 40 uL/mL Primary Hepatocyte Maintenance Supplements (Gibco)) is required and primary hepatocytes prefer maintenance supplements containing ITS, an insulin, transferrin, and selenium complex known as a hepatocyte growth factor [28].

Subsequently, the isolated hepatocytes were used to culture HEVs of different origins known for their association with HEV RNA, such as liver tissue samples and dried pork sausage samples. In the culture system we were able to show a reduction in the Ct value by comparison of the measured Ct values at D0 and D6 of the infection. This indicated an increase in HEV RNA, resulting from virus replication, thereby demonstrating the viability of infectious HEV present in the sample. Several HEV-3 liver samples from the field and HEV-3 and HEV-4 positive liver samples from HEV-infected animals were tested in the developed culture system. Replication was found for both zoonotic HEV genotypes and for different strains. A French sausage sample from a previous study described by Berto et al. [10] also showed replication in the primary hepatocyte cell culture system. However, the level of replication was not as high as found in some liver samples. This lower level of virus replication may be explained by the food processing procedure of the product or may be due to partial degradation of HEV particles due to the 10 years storage period (frozen at −80 °C) of these sausage samples.

The primary hepatocyte culture system did not show any cytopathic effect during the whole infection period. This is consistent with the previously mentioned cell lines, A549, PLC/PRF/5, and HepG2/C3A [16,17,18,19,20,26].

The undiluted (10^7^ genomic copies/mL) and 1000 times diluted (10^4^ genomic copies/mL) HEV virus was tested in the pig model (2 mL inoculation) as well as in in the hepatocyte cell culture model (1 mL inoculation). We were able to detect both HEV virus concentrations in both systems. In the cell culture model, we were even able to detect 2.5 × 10^3^ genome copies/mL of HEV. We did not test this concentration in the pig model. Bouwknegt et al., assessed the faecal–oral infection rate which more closely resembled the actual infection route and they found an infection rate of 1.4 × 10^6^ genomic copies/mL [29]. A similar outcome was reported by Andraud et al., where 10^6^ genomic copies of HEV gave an infection in 66% of the infected pigs; the infection doses of 10^4^ and 10^5^ genomic copies, showed no HEV infection [30]. Based on these results, the HEV infectivity model looks more sensitive than the pig model.

There are some limitations to the developed infectivity model. Firstly, the need for primary hepatocytes may be a limiting factor because access to fresh liver tissue will be required. If fresh liver is available, isolated primary hepatocytes can be conveniently stored in liquid nitrogen for later use. Primary hepatocytes could be immortalized to produce a cell line amenable to unrestricted passaging. However, it is not clear whether immortalization would result in a loss of cell properties needed for the HEV infection. Finally, in the developed method, PCR testing is required due to the absence of a cytopathic effect. In the future, the detection assay could be replaced by immunohistochemistry, to reduce the need for RNA extraction followed by real-time RT-PCR.

In summary, this study presents a primary hepatocyte-based cell culture system that was developed to demonstrate the infectivity of a range of Hepatitis E viruses in various food products. This method can be applied to test different food products to demonstrate the presence of infectious HEV and to better assess the risk of infection after consumption of food products associated with HEV RNA contamination.

## Figures and Tables

**Table 1:** Samples tested in the hepatocytes infectivity assay with the reference of their origin. Replicating virus was identified by demonstration of an increase in copy number per ml between D0 and D6 after inoculation.

Origin (Sample Type and HEV Genotype)	Log Genome Copies/mL of the Tested Sample	Country of Origin	Reference	Log Genome Copies/mL at D0	Log Genome Copies/mL at D6	Log Increase in Genome Copies/mL
Positive control (HEV PCR-positive hepatocytes HEV-3f)	6.42	The Netherlands	WBVR	1.99 (SD = 0.68)	5.44 (SD = 0.14)	3.45 (SD = 0.54)
Liver HEV-3f	5.10	The Netherlands	Bouwknegt et al. [22]	2.96 (SD = 0.68)	5.1 (SD = 0.23)	2.06 (SD = 0.91)
Liver HEV-4	3.66	Belgium	Hakze et al. [23]	1.29 (SD = 0.00)	2.51 (SD = 0.07)	1.22 (SD = 0.07)
Liver HEV-3f in different dilutions	7.37	Denmark	WBVR	See Table 2	See Table 2	See Table 2
Sausage	3.49	France	Berto et al. [10]	1.29 (SD = 0.00)	2.5 (SD = 0.00)	1.21 (SD = 0.00)
Faeces HEV-3c	6.49	The Netherlands	WBVR, 2019	3.58 (SD = 0.09)	4.67 (0.27)	1.1 (SD = 0.17)
Liver 2019A HEV-3c	7.16	The Netherlands	Boxman et al. [24]	3.14 (SD = 0.25)	4.04 (SD = 0.06)	0.63 (SD = 0.19)
Liver 2019B HEV-3c	6.39	The Netherlands	Boxman et al. [24]	3.04 (SD = 0.14)	3.52 (SD = 0.11)	0.48 (SD = 0.03)
Liver 2022A HEV-3c	4.39	The Netherlands	WFSR 2022	1.36 (SD = 0.10)	2.45 (SD = 0.07)	1.09 (SD = 0.03)
Liver 2022B HEV-3c	6.58	The Netherlands	WFSR 2022	2.64 (SD = 0.47)	3.16 (SD = 0.02)	0.52 (SD = 0.49)

## Data Availability

Not applicable.
